# Association of Long-Term Vascular Risk Factor Variability from Childhood and Midlife Cognitive Function: The Bogalusa Heart Study

**DOI:** 10.21203/rs.3.rs-9945900/v1

**Published:** 2026-07-09

**Authors:** Soo Jung Kang, Jeanette Gustat, Wan Tang, Phillip Hwang, Owen Carmichael, Ileana De Anda-Duran, Lydia Bazzano

**Affiliations:** Tulane University; Tulane University; Tulane University; Boston University; Pennington Biomedical Research Center; Tulane University; The University of Texas Southwestern Medical Center

**Keywords:** Vascular risk factor, variability, childhood, cognitive function

## Abstract

**Background:**

Variability in vascular risk factor (VRF) levels in adulthood has been associated with higher dementia risk. Whether VRF variability from childhood influences midlife cognitive function (CF) is unclear. We aimed to evaluate the association of long-term VRF variability from childhood with midlife CF, independent of mean levels.

**Methods:**

In this cohort study, participants in the Bogalusa Heart Study with ≥ 3 VRF (systolic blood pressure [SBP], fasting glucose, non-high-density lipoprotein cholesterol [non-HDL], and body mass index [BMI]) measurements from childhood and midlife CF assessment were included. VRF variability was measured using indices reflecting variability around the mean (SD, coefficient of variation [CV]) and around the age-adjusted slope (deviation from age-predicted values [DEV], residual SD [RSD]). CF was assessed using a global cognitive score (GCS) averaging eight standardized neuropsychological tests, and three cognitive domain performances. Associations between tertiles of VRF variability and CF were evaluated using multivariable linear regression with the lowest tertile as reference.

**Results:**

Among 1,010 participants (mean age 48 years, 61% women, 34% Black, mean follow-up 39 years), being in highest tertiles of SBP and glucose variability were associated with poorer GCS (SBP by DEV: β = −0.10, 95% CI = −0.17, −0.02; glucose by CV: β = −0.12, 95% CI = −0.20, −0.04), after adjusting for covariates. Being in middle and highest tertiles of SBP variability (by DEV) and glucose variability (by SD, CV) were associated with poorer episodic memory and executive function, with effect sizes increasing across higher degrees of variability. Association between non-HDL variability and CF was non-linear: only being in the middle tertile was associated with poorer executive function (by CV, β = −0.12, 95% CI = −0.23, −0.01). Association between BMI variability and CF was bidirectional across degrees of variability: being in the middle tertile of variability was associated with better GCS (by CV, β = 0.09, 95% CI = 0.02, 0.16); whereas being in the highest tertile (by DEV) was associated with poorer executive function, although attenuated after adjusting for APOE ε4 status.

**Conclusions:**

VRF variability from early life is a potential target for lowering risk of late-life cognitive dysfunction.

## Background

Midlife marks a period when individuals begin to show increasingly diverse cognitive trajectories.(1, 2) Because aging contributes to a wide range ofhealth outcomes including dementia, understanding midlife cognitive function (CF) can help distinguish age- related cognitive changes and pathological cognitive aging.(1) Understanding how heterogeneity of cognitive function (CF) in midlife is associated with risk factors of dementia may offer insight into pathways of cognitive aging.(1, 2)

The pathologic changes of dementia may begin many years before symptoms appear.(3)The WHO/Lancet Commission has recommended the implementation of a life course approach to prevent vascular cognitive impairment and dementia.(3) Because nearly half of dementia cases are associated with modifiable risk factors, therefore targeting modifiable risk factors such as vascular risk factors (VRFs) is a promising approach for lowering dementia case burden.(3) How long-term exposure to vascular risk factors (VRFs) from childhood can influence late life cognitive trajectories is explained by the “two-hit” vascular hypothesis.(4) Exposure to high levels of VRFs such as systolic blood pressure (SBP), glucose, lipids, and body mass index (BMI) from childhood can result in atherosclerosis and ischemia, leading to endothelial dysfunction of the blood brain barrier (BBB) (first hit) and subsequent brain amyloid beta and tau accumulation (second hit) as early as midlife.(4)

Levels of VRF change over time as physiologic regulatory mechanisms attempt to maintain homeostasis while responding to behavioral and environmental stressors. (5, 6) When this balance is disrupted, greater variability in VRFs may represent impaired homeostasis.(5, 6)

Importantly, relying solely on the mean levels of VRFs may overlook important information relevant to health risk, since VRF levels are not constant but fluctuate over the life course.(6) Even when average VRF levels fall within recommended ranges, individuals may still experience residual risk for cardiovascular disease, stroke, and mortality.(6) Evidence from adult populations indicates that greater variability in VRFs carries prognostic value for cardiovascular events, stroke, mortality, and dementia, independent of mean levels of VRFs.(7, 8) However, the extent to which long-term VRF variability from childhood shapes cognitive function (CF) in midlife is unclear.

The Bogalusa Heart Study(BHS) provides a unique opportunity to examine the association of long-term VRF variability from childhood on midlife CF, since it has prospectively collected repeated VRF measures from childhood.(9) Variability indices that account for nonlinear changes in VRF levels throughout childhood have been developed using the BHS data.(9, 10)Understanding how disruptions in VRF homeostasis from childhood across the life course influence midlife CF would contribute to prevention of late life cognitive dysfunction.(1) This study aimed to evaluate the association of long-term VRF variability from childhood on midlife CF using BHS data with the hypothesis that higher VRF variability from childhood is associated with poorer CF in midlife.

## Methods

### Study population and design

The BHS is a community based, longitudinal cohort study of VRFs from childhood to midlife that began in 1973 and continues to the present.(11) Between 1973 and 2016, the BHS conducted nine cross-sectional surveys of children aged 4–17 years, and those who were examined in childhood later participated in eleven cross-sectional surveys as adults. During the 2013–2016 examination cycle, 1,298 participants completed cognitive assessment. For the present longitudinal analysis, eligibility criteria included having at least three VRF measurements from childhood to midlife, a complete neuropsychological (NP) test battery at midlife, and complete covariates. After applying these criteria, the analytic sample consisted of 1,010 participants.

### General examination and laboratory analysis

Since 1973, standardized protocols have been employed by trained examiners across all study visits and procedures.(11) Participants were instructed to fast before examination for 12 to 14 hours.(12) Participants’ BP was measured on the right arm when relaxed and sitting by two trained examiners (each examiner obtained three replicates). The six BP measurements was averaged for analysis.(11) Glucose and cholesterol levels were determined from blood samples according to standardized protocols.(11) Non-HDL was calculated as total cholesterol minus HDL.(13) At each physical exam, height and weight were measured to the 0.1 cm and 0.1 kg in duplicate, and the duplicate measures were averaged for analysis.(11) Body mass index (BMI) was obtained as weight in kilograms divided by height in meters squared.

### Exposure: VRF variability

The main exposure was long-term variability in SBP, glucose, non-HDL, and BMI from childhood, assessed using four indices: SD, coefficient of variation (CV), deviation from age-predicted values (DEV), and residual standard deviation (RSD). Different types of variability indices were used, since no consensus exists for a gold standard approach to measuring VRF variability and different types of indices capture distinct aspects of variability.(7) SD and CV measure variability around the mean, while DEV and RSD capture variability around age-predicted slopes, accounting for nonlinear VRF trends.(7, 9) Age-predicted slopes of repeated measures of VRFs for each individual were estimated with linear mixed effect models (PROC MIXED, SAS, version 9.4) including random effects for race and sex, with age as the time scale. This approach allows for unequally spaced VRF measurements and nonlinear VRF change over time.(9, 10, 14) Final models included the following fixed effects; SBP (age, age^2^, age^3^), glucose and non-HDL(age and age^2^), and BMI (log(age)). For SBP, BMI, and non-HDL, random intercept and age were included as random effects. For glucose, only random intercept was added as random effects to achieve parsimony, as adding age did not improve model fit. BMI and non-HDL measures were logtransformed to address heteroscedasticity. Details on growth curve modeling and calculation of variability indices are provided in the supplemental methods. Each variability index was categorized into tertiles, with lowest, middle, and highest tertiles representing lowest, middle, and highest variability.

### Cognitive outcomes

CF was examined using the global cognitive score (GCS). The GCS, calculated by averaging standardized NP test scores into a single composite score, facilitates comparisons across studies.(15) The GCS was derived from eight standardized NP tests administered in the 2013–2016 examination by trained examiners.(16) In addition to the GCS, three cognitive domains were assessed following NIH toolbox guidelines, categorized to reflect the brain’s overall function, and to achieve brevity in cognitive assessment(17, 18): 1) executive function, using Trail- Making Test part B, and Digit Symbol Coding (WAIS-IV); 2) episodic memory, using Logical Memory I, Logical Memory II, (Wechsler Adult Intelligence Scale IV (WAIS-IV))(19) and Delayed Recognition (Wechsler memory scale-IV)(20); 3) attention and processing speed, using Digit Span Task Forwards (WAIS-IV) and Digit Span Task Backwards (WAIS-IV) and Trail-Making Test part A. Higher scores indicated better cognitive performance except for the Trail-Making Tests, which were reversed by subtracting each raw value from the maximum test score. Since cultural constructs may shape NP test performance and to address demographic differences in score distributions,, all NP test scores were standardized by age, race and sex to z-scores.(21) Age was standardized in 1-year increments. The final GCS was calculated by averaging the eight standardized NP test scores. Cognitive domain scores were computed by averaging standardized NP test scores within each domain.

### Covariates

Covariates were chosen based on their correlations with VRF variability or CF and were measured at the time of cognitive assessment.(9) Age, sex, and race were not included as covariates since NP test scores used to calculate the GCS were standardized by these variables.(16) Demographic and lifestyle information (age, race, sex, smoking status, educational level, employment status, and medication use) was obtained using validated questionnaires.(11) Cardiometabolic covariates were defined as: hypertension (SBP ≥ 130 mmHg or DBP ≥ 80 mmHg, or antihypertensive medication use), T2DM (fasting glucose ≥ 126 mg/dL or diabetes medication use), dyslipidemia (non-high-density lipoprotein cholesterol [non-HDL)] ≥ 130 mg/dL or lipid-lowering medication use), and obesity (BMI ≥ 30 kg/m^2^).(22) Non-HDL represents all atherogenic lipoproteins, calculated as total cholesterol minus HDL, and compared to LDL, is reported to be a better risk predictor of atherosclerotic cardiovascular events in individuals < 45 years. (23) The life’s Essential 8 has updated its lipid metric of interest to non-HDL since it can be reliably measured in the non-fasting state unlike traditional measures such as LDL.(24) Smoking status was categorized as current, former, or never. Life course mean VRF values were calculated as the average of repeated VRF measures and were entered as covariates to assess the independent effect of VRF variability on CF, since variability may increase as the mean values increase with age.(25) Employment status was classified as currently employed/not employed. Depressive symptoms were evaluated using the 10-item scale of Center for Epidemiologic Studies Depression (range 0–30, higher scores indicating greater depressed mood), with scores ≥ 10 representing depressed symptoms (sensitivity, 91.9; specificity, 92.8), and was dichotomized.(26) *APOE* ε4 carrier status was coded as a binary variable (0 = no ε4 allele, 1 = ≥ 1 ε4 allele). *APOE* ε4 status was inferred from two single nucleotide polymorphisms (SNPs), rs 429358 and rs7412, which were imputed using a reference panel. (The “rs” followed by a number is a unique identifier used to track and cross-reference SNPs.)(27, 28)Both genotyped and imputed methods for the two SNPs have been reported to be highly correlated (Cohen’s kappa fo rs 429358 and rs7412, 0.94 and 0.93).(29)

### Achieved education parameter

The quality of a person’s educational experience may not be fully reflected by the number of years of school education they completed.(21) Social determinants of health including race, income, and geographycan influence the quality of education, which is reflected in a person’s reading level. (21) In this study, since the reading level of a person strongly impacts NP test performance, an achieved education parameter was included as a covariate to better represent lifetime educational attainment.(21) This parameter was computed by averaging age-race-and sex-standardized scores from the word and letter reading tests (Wide Range Achievement Test 4 (WRAT-4)) and the vocabulary (WAIS-IV vocabulary subtest) test. (16, 21)

### Statistical analysis

Analyses were restricted to participants with complete data.. Participant characteristics were summarized using mean and SD or n (%). Differences in baseline characteristics by race and sex were evaluated using analysis of covariance with Tukey’s HSDfor pairwise comparisons, and categorical variables were compared using Pearson’s Chi-square test. Correlations among VRF variability indices were examined.(9)

Regression analyses were conducted with the GCS and each cognitive domain as separate outcomes. Each VRF variability measured by SD, CV, DEV, and RSD was entered into multivariable linear regression models as a categorical variable (tertiles), with lowest tertile of variability as reference. Previous studies have also categorized VRF variability into tertiles for easy interpretability.(30, 31)Furthermore, tertiles adequately represented the distribution of VRF variability in this study. Initially, models were unadjusted. For SBP variability, model 1 was adjusted for smoking, T2DM, obesity, dyslipidemia, and follow-up duration; model 2 additionally adjusted for life course mean SBP; model 3 further adjusted for achieved education parameter, employment, and depressive symptoms (SES); model 4 further adjusted for APOE ε4 status. For glucose variability, non-HDL variability, and BMI variability, the same models were applied as above, except model 1 adjusted for hypertension instead of T2DM/dyslipidemia/obesity, and model 2 adjusted for corresponding life-course mean VRF levels instead of SBP.

A two tailed p < 0.05 was considered significant. All statistical analysis was conducted by using SAS and R version 4.4.1. For brevity, only model 4 beta coefficients and 95% confidence intervals are reported in the Results section.

Sensitivity analyses by medication use were conducted separately for each VRF variability to examine potential residual risk of cognitive outcomes under clinical threshold levels of VRFs. Participants on medications that lowered the corresponding VRF levels were excluded. The same statistical approach as the main analysis was applied, using GCS as the outcome. For BMI variability, sensitivity analyses were performed by excluding antihypertensive, diabetes, and lipid-lowering medications one at a time. Because BMI variability can influence variability in other VRFs, and BP, glucose, and lipid levels typically fall during weight loss and rise during weight gain according to the repeated overshoot theory, these medications may affect the association between BMI variability and cognition.(32)

## Results

### Demographic and clinical characteristics

**Figure 1:**
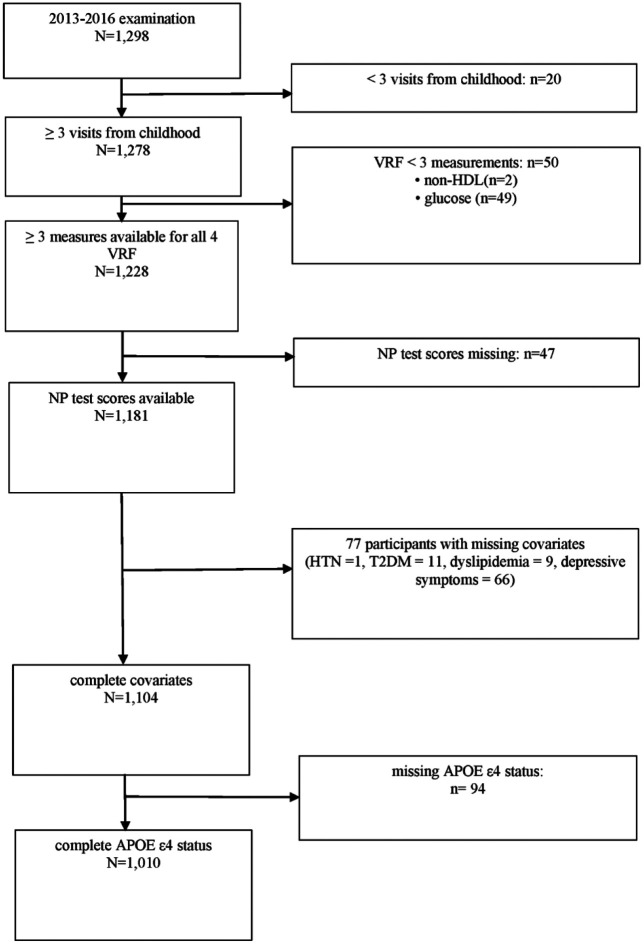
Flowchart of study participants Abbreviations: NP, neuropsychological; VRF, vascular risk factor

A total of 1,010 participants were included (Table 1). Mean age at cognitive assessment was 48 years (SD 5), mean follow-up was 39 years (SD 3), 61% were women, and 34% identified as Black. Participants completed a median of nine life-course visits (range 4–17). Mean life-course SBP, glucose, non-HDL, and BMI levels were 110 mmHg, 89 mg/dL, 119 mg/dL, and 24 kg/m^2^. Educational attainment, employment, and depressive symptoms differed by race and sex (all p <0.05). Participants excluded for missing *APOE* ε4 status were similar to those included (Figure 1, eTable 1).

**Figure 2:**
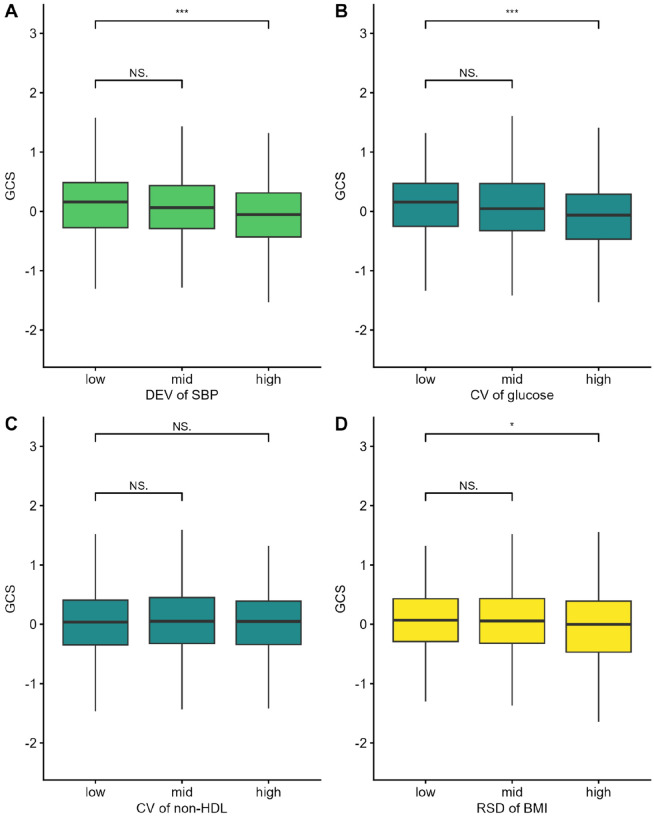
Distribution of global cognitive score across tertiles of vascular risk factor variability Abbreviations: SBP, systolic blood pressure; non-HDL, non-high density lipoprotein cholesterol; BMI, body mass index; GCS, global cognitive score; CV, coefficient of variation; DEV, deviation from age predicted values; RSD, residual SD; low, lowest tertile of variability; mid, middle tertile of variability; high, highest tertile of variability. *: p< 0.05, ***: p< 0.001

Since NP test scores were standardized by age, race, and sex, GCS and cognitive domain scores had a mean of 0 (eTable 2). Distribution of GCS across tertiles of vascular risk factor variability in representative variability indices is shown in Figure 2.

In representative variability indices shown in Figure 2, individuals in the top tertile of SBP by DEV, glucose by CV, and BMI variability by RSD had, on average, lower GCS than those in the bottom tertile (p <0.05). No differences in GCS were observed across tertiles of non-HDL variability by CV. Other variability indices showed similar patterns for SBP, glucose, and non-HDL; for BMI, other indices showed no difference in GCS across tertiles (data not shown).

**Figure 3:**
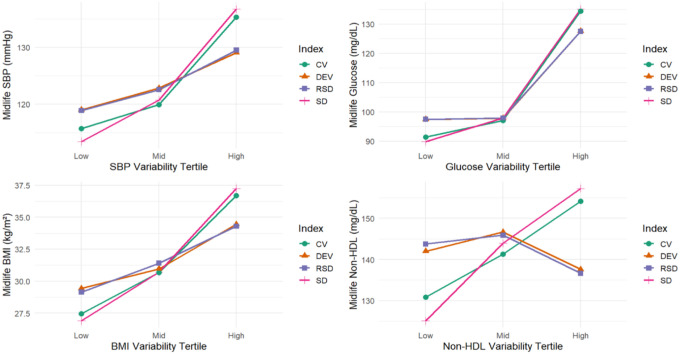
Midlife vascular risk factor levels by variability tertiles SBP, glucose, and BMI at midlife cognitive assessment were higher in individuals in the highest tertile compared with the middle or lowest tertiles across all variability indices (p < 0.001). Non-HDL at midlife cognitive assessment was higher in individuals in the middle tertile compared with the highest tertile when variability was measured by DEV (p = 0.013) or RSD (p = 0.011). Abbreviations: VRF, vascular risk factor; SD, standard deviation; CV, coefficient of variation; DEV, deviation from age predicted values; RSD, residual SD; low, mid, high, lowest, middle, highest tertile of VRF variability; SBP, systolic blood pressure; BMI, body mass index; non-HDL, non-high density lipoprotein cholesterol.

Midlife VRF levels by variability tertiles are shown in Figure 3.

Individuals in the top tertile of VRF variability had, on average, higher midlife VRF levels than those in the middle and bottom tertiles, except for non-HDL variability. Patterns of midlife non-HDL levels differed by variability index. When non-HDL variability was measured by DEV or RSD, individuals in the middle tertile had higher midlife non-HDL levels than those in the top tertile (by DEV, p = 0.013; by RSD, p = 0.011). BMI and non-HDL variability showed modest correlations with SBP and glucose variability (eTable 3).

### VRF variability and GCS

After adjusting for smoking, other VRFs, follow-up duration (model 1), life course mean SBP or glucose levels (model 2), SES (model 3), and APOE ε4 status (model 4), compared with the lowest tertile, being in the highest tertile of SBP and glucose variability was associated with poorer GCS (SBP by DEV: β = −0.10, 95% CI = −0.17, −0.02; glucose by SD: β = −0.11, 95% CI = −0.19, −0.03; glucose by CV: β = −0.12, 95% CI = −0.20, −0.04) (Table 2). BMI variability showed bidirectional associations with GCS by degree of variability. Being in the middle tertile of BMI variability was associated with better GCS (model 4)(by SD: β = 0.1, 95% CI = 0.03, 0.17; by CV: β = 0.09, 95% CI = 0.02, 0.16). In contrast, being in the highest tertile of BMI variability by DEV and RSD was associated with poorer GCS, but was attenuated after adjusting for life course mean BMI (model 2). No associations between non-HDL variability and GCS were observed.

### VRF variability and cognitive domains

Compared with the lowest tertile, being in middle and highest tertiles of SBP variability (by DEV) and glucose variability (by SD, CV) were associated with poorer episodic memory and executive function, showing a graded pattern in which effect sizes increased with higher degrees of variability (model 4; Table 3–5). BMI variability again showed bidirectional associations with cognitive domain performances by degree of variability. Being in the middle tertile of BMI variability was associated with better executive function (by SD) and attention and processing speed (by SD, CV, DEV)(model 4). In contrast, being in the highest tertile of BMI variability was associated with poorer executive function (by DEV, RSD), although these associations were attenuated after adjusting for SES (model 3, by RSD) or APOE ε4 (model 4, by DEV). For non-HDL, only being in the middle tertile of variability (by CV) was associated with poorer executive function (model 4); being in the highest tertile was not associated with cognitive domain performances.

When variability indices were considered, all four indices captured the associations between being in the highest tertile of SBP and glucose variability and midlife CF (eTable 4). For BMI, only DEV and RSD captured associations between being in the highest tertile of variability and midlife CF. For non-HDL, only CV captured the association between being in the middle tertile of variability and midlife CF.

### Sensitivity analysis by medication use

Excluding participants on medication produced similar results: higher SBP and glucose variability remained associated with poorer GCS, and higher BMI variability with better GCS. Non-HDL variability remained unassociated with GCS (data not shown).

## Discussion

In this community-based cohort, long-term variability in SBP, glucose, non-HDL, and BMI from childhood was associated with midlife cognitive function, independent of mean levels and relevant covariates. As hypothesized, higher SBP and glucose variability were consistently linked to poorer global cognition and to poorer episodic memory and executive function, with progressively larger effect sizes with increasing degrees of variability. Association between BMI variability and midlife cognitive function showed a bidirectional pattern: having moderate variability was associated with better cognitive function, whereas having highest variability was associated with poorer cognitive function, suggesting a potential BMI threshold. Association between non-HDL variability and midlife cognitive function was non-linear: only moderate variability was associated with poorer executive function. Sensitivity analyses excluding participants on vascular risk factor-lowering medications yielded similar results, supporting the robustness of these findings.

Our study extends the exposure window beyond previous studies on long-term VRF variability by evaluating VRF variability from childhood through midlife. Our findings are consistent with those of the CARDIA study, where greater variability in BP and glucose from young adulthood was linked to poorer midlife CF.(33, 34) Our study also examines BMI and non-HDL variability from childhood in relation to midlife CF. Prior studies of BMI and lipid variability and cognition have largely involved older adults. Although variability in other lipid fractions and cognition have been examined in older adults, few studies have focused on non-HDL variability from childhood, which may offer a more comprehensive insight into the link between lipid variability and cognition. In nondemented participants > 70 years in Alzheimer’s Disease Centers, higher BMI variability was associated with faster cognitive decline over five years.(35) In the Aspirin in Reducing Events in the Elderly (ASPREE) trial, greater variability in total and low-density lipoprotein cholesterol in adults > 70 years predicted increased risk of dementia and cognitive decline over five years. (36) Cognitive trajectories may start to diverge as early as the fourth decade.(2)Although our study assessed cognition onlyonce in midlife, evaluating risk factors that may shape midlife cognition such as VRF variability from childhood may help identify when adverse influences first emerge and define the optimal window for intervention in cognitive disorders.(1)

A steady supply of oxygen and nutrients is essential for brain health.(37) Common mechanisms through which long-term VRF variability from childhood may affect midlife CF include oxidative stress and inflammation.(4) Increased SBP variability can promote brain vascular injury via shear stress and recurrent hypoperfusion, contributing to hippocampal and white matter ischemia and repeated vascular remodeling.(37) The resulting neuronal loss may further impair autonomic regulation, reinforcing SBP variability.(37) Glucose variability can trigger recurrent hyper- and hypoglycemic episodes that disrupt cerebral blood flow and contribute to vascular and gray matter injury.(38) Glucose variability may also impair insulin signaling at the BBB and promote central insulin resistance.(39) Mechanisms linking non-HDL variability to cognition are less clear. Non-HDL variability could be an epiphenomenon reflecting the severity of other VRFs and underlying disturbances in homeostasis.(36) Alternatively, non-HDL variability may contribute to atherosclerotic plaque instability, potentially increasing risk of cerebrovascular events. (31)

BMI variability (weight cycling) may influence cognition through the following pathways.(8) The visceral fat repartition hypothesis proposes that weight cycling redistributes body fat preferentially to the abdominal region; with loss of thigh fat and muscle during weight-loss and preferential central fat gain during weight regain. Visceral fat is metabolically active and may contribute to adverse lipid profiles, insulin resistance, and impaired insulin transport across the BBB.(8, 40, 41) Fluctuations in adipocyte - derived neuroprotective hormones such as leptin may also affect CF.(42) The “repeated overshoot” hypothesis proposes that BMI variability may induce variability in other VRFs, with BP, glucose, and lipid levels falling during weight loss and rising during weight gain, resulting in repeated metabolic stress that cumulatively affects brain health.(32)

In our study, a non-linear association was observed between non-HDL variability and midlife CF. Compared with the lowest tertile, being in the middle tertile of non-HDL variability measured by CV was associated with poorer executive function, whereas being in the top tertile showed no association. Individuals in the top tertile of non-HDL variability by CV also had the highest non-HDL levels at midlife. Our findings suggest a threshold saturation effect; when non-HDL levels increase over the life course and reach a threshold, additional variability may no longer influence cognition. A threshold effect between non-HDL and cognition has been reported in older adults, underscoring the complexity of this relationship. In middle aged and older adults in the China Health and Retirement Longitudinal Study (CHARLS), increasing non-HDL was initially associated with lower cognitive risk, but beyond an inflection point, risk increased with higher non-HDL levels.(43) Elevated midlife non-HDL levels among individuals in the top tertile of variability in our study may reflect cumulative exposure to increased non-HDL variability from early life.(44) Monitoring non-HDL variability as well as mean non-HDL levels from early life may help detect residual cognitive risk before dyslipidemia becomes clinically apparent.

The bidirectional association between BMI variability from childhood and midlife CF suggests a potential threshold effect. In our study, individuals in the middle tertile of BMI variability by SD or CV had lower midlife BMI than those in the top tertile, whose average BMI exceeded 35 kg/m^2^ at midlife. Moderate BMI variability may be protective, whereas once BMI reaches a higher level, additional variability may become harmful. Although nonlinear associations between BMI and cognition have been reported in adults, the BMI inflection point at which risk shifts from protective to detrimental from early life remains poorly defined. Only one study has reported a BMI inflection point of 23 kg/m^2^ in midlife adults with high cardiovascular risk.(45) Several factors may explain the apparent benefit of moderate BMI variability from childhood on midlife CF. Because BMI reflects muscle mass as well as adiposity, moderate BMI variability may also represent increases in muscle mass.(46) In a population with a high VRF burden such as the BHS, moderate BMI variability may also reflect intentional weight loss from healthy lifestyle behaviors. According to the repeated overshoot theory, BMI variability can influence variability in other VRFs.(32) Individuals in the middle tertile of BMI variability may be engaging in healthy lifestyle behaviors to lose weight, which may also lower SBP and reduce SBP variability, supporting better midlife CF. Conversely, in our study, being in the highest tertile of BMI variability was associated with poorer midlife CF compared with the lowest tertile, although these associations were attenuated after adjusting for life course mean BMI, SES, and APOE ε4 status, suggesting that part of the BMI threshold effect may reflect these factors. Individuals in the highest tertile of BMI variability may also have a greater VRF burden, and APOE ε4 may act synergistically with VRFs to adversely impact cognition, potentially masking the independent contribution of BMI variability.(47)

Different variability indices may be more informative depending on context, as they capture distinct aspects of variability.(7) Associations between being in the highest tertile of BMI variability from childhood and midlife CF was captured only when variability was measured by DEV or RSD, suggesting these indices may be more reliable than SD or CV at higher BMI levels. Because DEV and RSD measure variability around the age-adjusted mean, they are less influenced by extreme VRF levels across the life course than SD or CV.(48) Conversely, if a threshold non-HDL level exists above which variability no longer influences CF, CV may be the most appropriate index for detecting associations at lower non-HDL levels. In our study, only CV captured the association between being in the middle tertile of non-HDL variability and poorer midlife executive function compared with the lowest tertile, likely because CV partially adjusts for mean non-HDL across the life course. Individuals in the middle tertile of non-HDL variability had the lowest midlife non-HDL levels when variability was measured using CV, enabling detection of associations independent of mean levels.(5) Notably, midlife non-HDL was lowest in individuals in the top tertile of non-HDL variability when variability was measured using DEV and RSD, a pattern that may reflect reductions in non-HDL across the life course through healthy lifestyle behaviors.

VRFs are known to be intercorrelated, and share mechanisms such as atherosclerosis, chronic inflammation and oxidative stress, contributing to adverse health outcomes.(49) In this study, all four VRF variability were positively correlated with one another. Our findings suggest high variability in ≥ one VRF from childhood often coexist and could synergistically influence midlife CF over the life course (eFigure 1). As explained by the repeated overshoot theory and visceral fat repartition theory, BMI variability together with non-HDL variability may influence SBP variability and glucose variability over the life course, which are more downstream processes. (32) The resulting higher long-term SBP and glucose variability over the life course in response to increased long-term BMI and non-HDL variability from childhood could directly contribute to poorer midlife CF.

Strengths of this study include the longitudinal examination of four VRF variability from childhood throughout the life course using different types of variability indices, which effectively captured the non-linear associations between BMI and non-HDL variability and midlife CF. Our study has several limitations. First, CF was measured once in midlife, therefore subsequent worsening of cognition could not be assessed, potentially biasing results toward the null. Second, other adiposity measures such as weight circumference were not evaluated.(46) Third, the directionality of VRF variability was not accounted for in our study. Fourth, as an observational study, causal inference is not possible. Fifth, measurement error or lack of data on physical activity, childhood diet, or medication adherence may have resulted in residual confounding. Sixth, the BHS participants are at a relatively young age and from a single geographic location, which may limit generalizability.

## Conclusions

In conclusion, higher long-term SBP and glucose variability from childhood were associated with poorer midlife cognitive function. Long-term BMI and non-HDL variability from childhood may contribute to higher SBP and glucose variability over the life course and may synergistically influence midlife cognitive function. Targeting vascular risk factor variability from early life may help prevent late-life cognitive dysfunction.

## Supplementary Material

Tables 1 to 5 are available in the Supplementary Files section.

Supplementary Files

This is a list of supplementary files associated with this preprint. Click to download.

- SupplementalMaterialsBMCSJK0605.docx
- Tables.docx
- Additionalmaterials.docx

## Data Availability

Data from the Bogalusa Heart Study used in this analysis are not publicly available due to institutional data-use agreements. De-identified data may be made available from the corresponding author (SJK, skang4@tulane.edu) upon reasonable request and approval from the Bogalusa Heart Study Steering Committee.

## Abbreviations

BHS: Bogalusa Heart Study
BMI: body mass index
CF: cognitive function
CV: coefficient of variation
DEV: deviation from age-predicted values
GCS: global cognitive score
Non-HDL: Non-high density lipoprotein
NP: neuropsychological
RSD: residual standard deviation
SD: Standard deviation
VRF: Vascular risk factor
